# Morphological and physiological responses of *Dalbergia odorifera* T. Chen seedlings to different culture substances

**DOI:** 10.1371/journal.pone.0232051

**Published:** 2020-05-20

**Authors:** Xiao-Hui Yue, Ling-Feng Miao, Fan Yang, Mohsin Nawaz

**Affiliations:** 1 College of Ecology and Environment, Center for Eco-Environmental Restoration Engineering of Hainan Province, Key Laboratory of Agro-Forestry Environmental Processes and Ecological Regulation of Hainan Province, Hainan University, Haikou, Hainan, China; 2 Research Center for Eco-Environmental Sciences, Chinese Academy of Sciences, Beijing, China; 3 College of Plant Protection, Hainan University, Haikou, Hainan, China; 4 Key Laboratory of Genetics and Germplasm Innovation of Tropical Special Forest Trees and Ornamental Plants, Ministry of Education, College of Forestry, Hainan University, Haikou, Hainan, China; Chinese Academy of Forestry, CHINA

## Abstract

*Dalbergia odorifera* T. Chen seedlings do not grow well in the typical red soils of tropical regions. Eighteen culture substances filled with different substrate combinations and proportions of red soil, coconut coir powder, deciduous leaf powder, and sand were used as to determine their effects on the growth, root system development, dry matter accumulation and allocation, leaf relative electrolyte leakage, chlorophyll content, root superoxide dismutase activity, root malondialdehyde content, and total soluble sugar content of *D*. *odorifera*. Results demonstrated that different substrate combinations and proportions had different effects on the performance of *D*. *odorifera*. All mixed substrates were better than any single substrate. The suitable substrate combinations and proportions of sand, coconut coir powder, and deciduous leaf powder mixed with red soil improved the growth, root architecture, and physiological characteristics of *D*. *odorifera* seedling. For example, groups C1_-2_ (coconut coir/red soil = 2/2, v/v, the same below) and C3_-2_ (red soil/sand = 2/2) exerted the best effects on plant growth and biomass accumulation. Groups C1_-2_, C2_-2_ (deciduous leaf powder/red soil = 2/2), and C3_-2_ remarkably enhanced root system development. Group C6 (coconut coir/red soil/sand = 1/1/1) substantially promoted root nodule development. Group C3_-1_ (red soil/sand = 3/1) exhibited the best effects on physiological characteristics. On the basis of the comprehensive evaluation of Euclid’s multidimensional space mathematical model, we found that the suitable substrate combinations followed the order of C1_-2_ > C3_-1_ > C2_-2_. This research provides scientific guidance for the proper seedling culture of *D*. *odorifera* and the rational utilization of solid wastes such as coconut coir and deciduous leaves of *Ficus elastica*.

## Introduction

*Dalbergia odorifera* T. Chen, also named yellow flower pear, is a species that belongs to Family Leguminosae. This tree can reach 10–15 m in plant height [1]. *D*. *odorifera* is a fragrant rosewood species under the second-grade state-protection of the Chinese government [2, 3]. This species is naturally distributed in the tropical regions of China, especially in Hainan Province [1]. *D*. *odorifera* has abundant secondary plant metabolites that have antibacterial, anti-inflammatory, antioxidative, antithrombotic, antiosteoporosis, antiangiogenesis, antiosteosarcoma, and antiplatelet activities. Therefore, *D*. *odorifera* species is widely used as a medicinal drug for the treatment of various illnesses, such as cancer, cardiovascular diseases, blood disorders, necrosis, diabetes, and rheumatic pain [3, 4]. *D*. *odorifera* was introduced in subtropical areas in China and has been widely cultivated in recent decades due to its high medicinal and economic value [4]. However, *D*. *odorifera* seedlings cultivated in typical tropical red soil exhibit poor plant growth and root architecture due to the soil’s low-porosity and pH value, and high ferrum and aluminum ionic contents [5]. This condition results in low survival rates when seedlings are transplanted. Hence, the growth and root system development of *D*. *odorifera* seedlings must be improved through the use of appropriate culture matrixes.

In recent years solid wastes, such as cottonseed hulls, sawdust, carbonized rice coirs, sugar slag, sludge, almond shells, and pineal compost, are increasingly being utilized as seedling culture substrates to promote plant growth [6–8]. Coconut (*Cocos nucifera* Linn.) coir and the fallen leaves of *Ficus elastica* Roxb. ex Hornem, are very common and typical solid wastes in Hainan Province. Coconut coir may improve the growth of *Lycopersicum esculentum* and *Capsicum annuum* when used as a substrate [9, 10]. The fallen leaves of sycamore and aspen may enhance soil structure, soil organic matter content and fertility status thus plant growth may be promoted by deciduous matrixes [11]. Several studies have demonstrated that mixed matrixes are better for plant development because they improve soil aeration, water retention capacity, and soil nutrients than any single matrix [9–14]. For example, the growth behavior of tomato and marrow squash seedlings is directly related to the composition of seedling substrate. Decomposed vinasse, mushroom residue, and cattle manure can improve the total primary nutrient condition of cultivation matrixes and further enhance the stem diameter, dry weight accumulation, and health index of the tested seedlings [12]. The mixture of perlite and rice coir biochar as a hydroponic substrate for growing cabbage, dill and red lettuce is better than a single substrate treatment, and has the advantage of increased yield [13]. In addition, waste wood, pine bark, sand and volcanic rock have been used as single or compound substrates for the cultivation of commercial vegetable seedlings [14].

Few studies have reported that temperature affects the germination rates of *D*. *odorifera* seeds, and lighting spectra and exogenous substances influence the growth and nutrient absorption capacity of its seedlings [15, 16]. Intraspecific geographic variations in the optimal germination temperature have been noted in *D*. *odorifera* seeds collected from four geographic sites in southern China; the optimal germination temperature for the seeds is reportedly within the range of 25 °C–30 °C [15]. The exogenous application of chitosan oligosaccharide (1/800, v/v) under 280 W light-emitting diode panels for a 15 h daily photoperiod can efficiently promote the synthetic quality and nutrient utilization of *D*. *odorifera* seedlings [16]. However, few studies have been conducted on the culture substrates of *D*. *odorifera* seedlings, and the influence of matrix on seedling growth and root development has been rarely explored. Root systems are the important bridge between soil and plant, and play a vital role in the life cycle of plants, especially for plants that need to be transplanted [17, 18].

Different substrate combinations and proportions from coconut coir powder, deciduous *F*. *elastica* leaf powder, local river sand, and red soil were used as culture substrates to determine their effects on the growth, root system development, and physiological characteristics of *D*. *odorifera*. This study aimed to answer the following questions: (1) Can solid wastes such as coconut coir powder and deciduous *F*. *elastica* leaf powder, be used as substrate for *D*. *odorifera* seedlings? (2) How will seedling growth and root systems of *D*. *odorifera* respond under various substrate combinations in different proportions? (3) Which substrate combinations and in what proportions are suitable for plant growth, root system and root nodule development, leaf pigment synthesis, and root physiobiochemical characteristics? We hypothesized that the growth, root system development, and physiological characteristics of *D*. *odorifera* differentially respond to the same optimal culture substances.

## Materials and methods

### Plant material and experimental setup

Mature and healthy *D*. *odorifera* seeds were collected from the plantation forest at Ledong County, Hainan Province (18° 42′ 57.91″ N, 108° 52′ 18.65″ E) on Februrary 10, 2017. Although *D*. *odorifera* belongs to protected plant species, the trade of its commercialized seeds and seedlings is permitted and legal in China. Therefore, we confirmed that no specific permissions for the seed collection in these locations were required by Forestry Bureau of Hainan Province, China. The seeds were stored at 4 °C in a refrigerator. The seeds were placed in an artificial climate incubator for germination at 27 °C and 85% humidity in light for 12 h and 24 °C and 75% humidity in the dark for 12 h on September 11, 2018. After germinating and growing, the seedlings with the same size were transplanted in pots (20 cm diameter and 15 cm height) on October 10, 2018. Two seedlings were planted in each pot. The pots were filled with equal volumes of culture substrates from coconut coir powder, deciduous *F*. *elastica* leaf powder, river sand and red soil according to the following experimental designs. Powder size was less than 5 mm. The pots with transplanted seedlings were placed in the greenhouse with natural light source in Hainan University, Haikou, Hainan Province (20° 03′ 26.77″ N, 110° 19′ 0.90″ E). Average air temperature was 22 °C– 28 °C, average air humidity was 42%– 85%, average light intensity was 3200–6500 Lux in daytime.

A random block design was employed. The substrate combinations were prepared as follow. C1 group had three subgroups with coconut coir powder and red soil (C1_-1_: 3/1; C1_-2_: 2/2; C1_-3_: 1/3, v/v), C2 group had three subgroups with deciduous leaf powder and red soil (C2_-1_: 3/1; C2_-2_: 2/2; C2_-3_: 1/3, v/v), C3 group had three subgroups with red soil and sand (C3_-1_: 3/1; C3_-2_: 2/2; C3_-3_: 1/3, v/v); C4 group had three subgroups with coconut coir powder and sand (C4_-1_: 3/1; C4_-2_: 2/2; C4_-3_: 1/3, v/v); C5 group contained coconut coir powder, deciduous leaf powder and red soil (1/1/1, v/v); C6 group contained coconut coir powder, red soil, and sand (1/1/1, v/v); C7, C8, C9, and C10 groups were entirely constituted of coconut coir powder, red soil, deciduous leaf powder, and sand, respectively. The pots were watered until 100% field capacity every day, and excess water in the dish placed under the pot was re-watered to the corresponding pot to prevent nutrition loss. Six replicates with six seedlings each were used for each group or subgroup. Plant height, leaf area, and number of leaf were determined after 60 days growth. Fresh leaves and root samples for physiobiochemical analysis were collected and immediately frozen in liquid N. Fresh leaves, shoots, and roots were individually harvested for biomass and image analyses.

### Determination of plant growth, dry matter accumulation and allocation, and root system development

The height of *D*. *odorifera* seedlings was determined with a ruler with a precision of 0.1 cm. Leaf area was measured by an LI-3000C portable leaf area meter. The total number of leaves was counted. All seedlings were harvested at the end of the experiment, and divided into leaves, shoots, and roots. The roots were washed, dried in the shade, and scanned with a Perfection V700 photo color image scanner. Total root length, entire surface area, total volume, average diameter, number of root tips, and number of branches were analyzed via the Winrhizo system. Biomass samples were dried (at 70 °C for 48 h) until constant weight, and specific leaf area (SLA) was then calculated by dividing the leaf area by the leaf dry weight according to Xu et al. [19]. Dry matter allocation was calculated according to the ratio of root to the sum of leaves and shoots [20, 21].

### Determination of leaf relative electrolyte leakage and pigment content

In brief, five freshly harvested leaf disks (0.5 cm in diameter) were placed in tubes containing 10 mL of deionized water and incubated at 25 °C on a shaker for 6 h. Then, the initial electrical conductivity was determined using a conductivity meter (Mettler-Toledo Instruments Co., Ltd, Shanghai, China). The final conductivity was measured after boiling at 100 °C for 30 min using previous samples. Relative electrolyte leakage (REL) was determined as the ratio of the initial conductivity to final conductivity [22]. Chlorophyll pigments were extracted in 80% (v/v) chilled acetone and quantified using a spectrometer (MPDA-1800, Shanghai, China). The absorption spectra of the samples were recorded at 663, 647, and 470 nm wavelength for chlorophyll a, chlorophyll b, and carotenoids, respectively. The absorbance values were converted to concentrations and/or contents according to the experimental equations described by Lichtenthaler [23].

### Estimation of root malondialdehyde, superoxide dismutase, and soluble sugar contents

Fresh root samples (0.3 g) were homogenized in 8 mL 5% trichloroacetic acid (TCA) solution and centrifuged at 10,000 rpm for 10 min, thiobarbituric acid (2 mL. 0.6%) in 10% TCA was added to 2 mL of the supernatant. The mixture was boiled at 100 °C for 30 min. Then, the absorbances of the supernatant at 450 (A_450_), 532 (A_532_) and 600 nm (A_600_) was determined with a MAPDA spectrometer. Malondialdehyde (MDA) concentration was calculated through the following formula: C (μmol/L) = 6.45 × (A_532_ − A_600_)– 0.56 × A_450_ [24]. Soluble sugars were extracted and spectrophotometrically estimated using an anthrone sulphuric acid reagent according to the method of Xiao et al. [22] and Renaut et al. [25]. Superoxide dismutase (SOD) was extracted with sodium phosphate buffer containing 50 mM sodium phosphate buffer (pH 7.8), 1 mM ethylenediamine tetraacetic acid, 15% glycerin, 1 mM ascorbic acid, 1 mM dithiothreitol, 1 mM glutathione, 5 mM MgCl_2_, and 1% (w/v) polyvinylpolypyrrolidone [26]. SOD activity was measured spectrophotometrically at 560 nm by monitoring the inhibition of the photochemical reduction of nitroblue tetrazolium according to the method of Xiao et al. [22].

### Comprehensive evaluation based on the multidimensional space mathematical model of Euclid

The responses of *D*. *odorifera* to different culture substrate compositions based on a single and independent parameter are difficult to evaluate. However, the multidimensional space (Euclid, E^n^) mathematical model is a useful tool to comprehensively assess the effects of various culture substrate compositions on the above- and below-ground growth and development, and physiological responses of *D*. *odorifera*. This model can be calculated by using following equation as reported by Wang et al. [27]:

$$\sum {\text{P}}_{i}{}^{2}=\sum {\left(1-{\text{a}}_{ij}\right)}^{2},$$

where P_*i*_ stands for the distance from the *i*-th treatment to the standard point, and the performance of treatment is better when the value of P_*i*_ is smaller; *i* represents treatment; *j* stands for the trait, and a_*ij*_ represents the absolute value of the ratio of *j* trait of the *i*-th treatment to the maximum value of the *j*-th trait (the minimized value is selected under negative correlated condition).

### Statistical analysis

Results were expressed as means ± standard errors (n = 6). SPSS 13.0 software package was used for statistical analysis. One-way ANOVA followed by Duncan's multiple range test at P < 0.05 was employed to assess the statistical significant difference between treatments.

## Results

### Morphological variations

**Fig 1:**
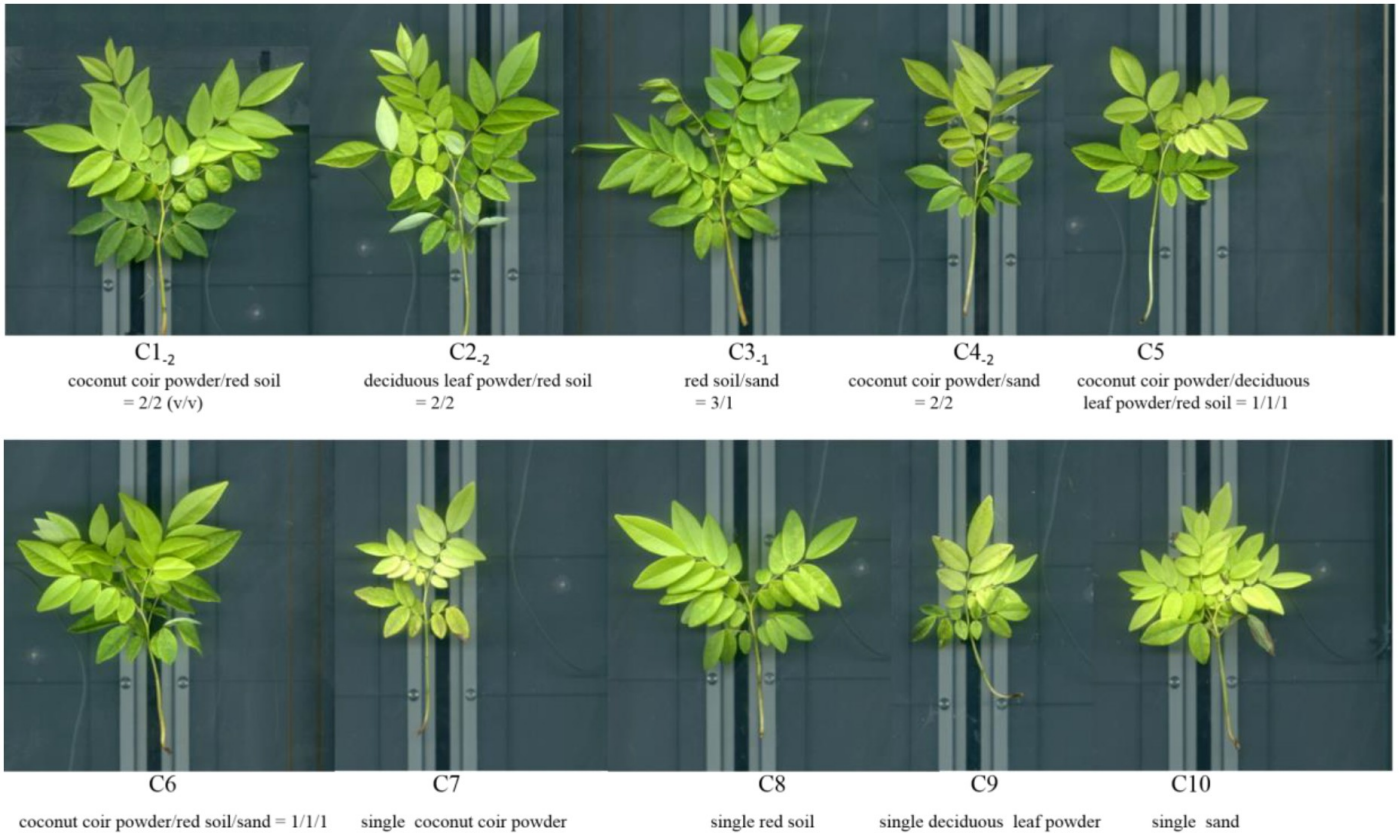
Above-ground morphological traits of *D*. *odorifera* cultured by different substrates.

**Fig 2:**
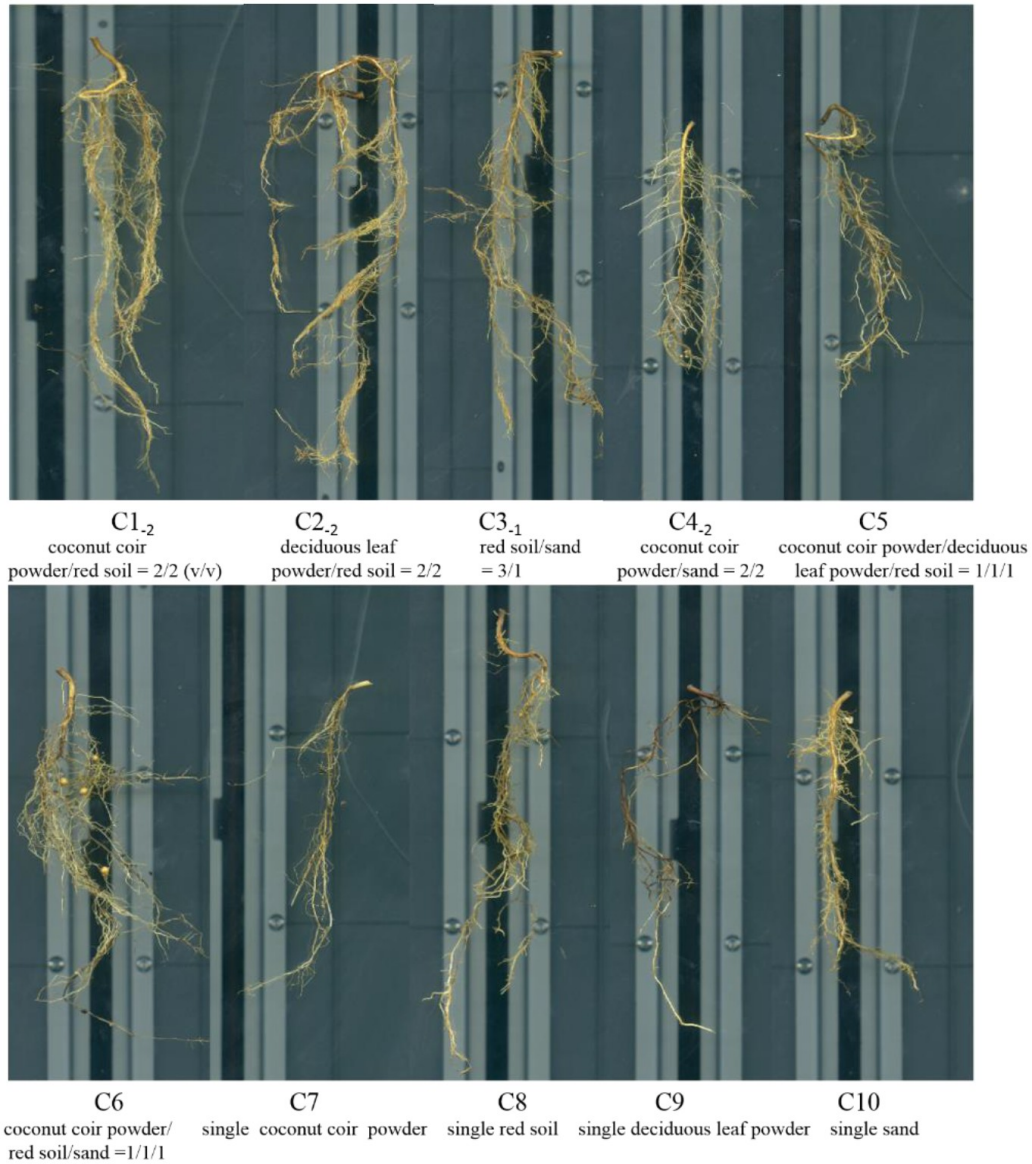
Below-ground morphological traits of *D*. *odorifera* cultured by different substrates.

The composition of different substrates had remarkable effects on the above-ground and below-ground growth and development of *D*. *odorifera*, as shown in the Figs 1 and 2, respectively. In general, seedlings from the C1_-2_, C2_-2_, C3_-1_, and C6 groups had a larger crown width, more leaves and branches, and dark green in color compared with those in the other groups. Seedlings from the C7, C9, and C10 groups were remarkable shorter, smaller, and yellower than the seedlings described above (Fig 1). The root architecture of *D*. *odorifera* from the C1_-2_, C2_-2_, C3_-1_, C6, and C8 groups were well developed, and several fine root hairs and branches or long roots were observed. The root system from the C7 and C9 groups had the worst performance in comparison with the others. Interestingly, several visible root nodules could be found in the C6 group but not in other groups (Fig 2). Thus, culture substances consisting of sand, coconut coir powder, and red soil could improve the whole performance of *D*. *odorifera* seedlings.

### Effect of substrate combinations and proportions on the seedling growth of *D*. *odorifera*

Significant changes (P < 0.05) in plant height, leaf area, number of leaves, and total leaf area were observed among groups from different substrate combinations and proportions (Table 1). In general, the mixed substrates might efficiently promote the growth of *D*. *odorifera* seedlings than any single substrate. Seedlings from the C6 group filled with equal volumes of coconut coir powder, red soil, and sand achieved the highest plant height and largest average leaf area compared with those in other groups. Seedlings from the C3 group filled with red soil and sand had the most leaf numbers, the largest total leaf area, and a considerable higher plant height than those in other groups. Different substrate proportions from different subgroups caused significant differences (P < 0.05) in plant height, leaf area, number of leaves, and total leaf area within the same group with the same substrate composition. Overall, the C1_-2_, C2_-2_, and C3_-1_ subgroups had better performances than the other subgroups within the same group. Interestingly, the single red soil had a balanced and better performance in plant height, leaf area, number of leaves, and total leaf area even when compared with some groups filled with mixed substrates. The single substrate of coconut coir, deciduous leaf powder, and sand (especially deciduous leaf powder and coconut coir) remarkable inhibited the growth and development of *D*. *odorifera* seedlings. Thus, culture substances consisting of sand, coconut coir powder, and red soil could improve the growth of *D*. *odorifera* seedlings. The C1_-2_ and C3_-2_ subgroups had the best effects on plant growth.

**Table 1:** Effect of various substrate combinations and proportions on the growth of *D*. *odorifera* seedlings. Different lowercase letters in the same column indicate the significant difference among different substrate combinations (P < 0.05). Different uppercase letters in the same column indicate the significant difference among the same substrates with different proportions (P < 0.05).

Treatment group	Height (cm)	Average leaf area (cm^2^)	Number of leaves	Total leaf area (cm^2^)
**C1 (Coconut coir powder**: **red soil)**	**12.7 ± 0.4 b**	**3.17 ± 0.18 bc**	**27 ± 1 de**	**86.7 ± 3.6 c**
C1_-1_ 3: 1	11.2 ± 0.8 C	3.11 ± 0.27 B	24 ± 1 B	75 ± 5.5 B
C1_-2_ 2: 2	14.2 ± 0.5 A	3.90 ± 0.73 A	33 ± 3 A	128.6 ± 18.7 A
C1_-3_ 1: 3	12.8 ± 0.7 B	2.49 ± 0.25 B	25 ± 2 B	62 ± 8.5 C
**C2 (Deciduous leaf powder: red soil)**	**12.7 ± 0.8 b**	**2.98 ± 0.21 c**	**30 ± 1 cd**	**88.9 ± 5.6 c**
C2_-1_ 3: 1	11.7 ± 1.1 B	1.75 ± 0.19 C	24 ± 2 B	42.9 ± 6.9 C
C2_-2_ 2: 2	13.4 ± 0.9 A	3.30 ± 0.20 B	33 ± 3 A	109.5 ± 11.2 B
C2_-3_ 1: 3	12.9 ± 1.2 A	3.88 ± 0.36 A	32 ± 5 A	124.3 ± 17.4 A
**C3 (Red soil: sand)**	**13.5 ± 0.7 ab**	**2.92 ± 0.2 c**	**39 ± 2 a**	**113.6 ± 7.6 a**
C3_-1_ 3: 1	13.4 ± 0.5 B	3.67 ± 0.24 A	38 ± 5 A	140.2 ± 16.6 A
C3_-2_ 2: 2	14.5 ± 1.1 A	2.68 ± 0.09 B	41 ± 3 A	110.7 ± 6.8 B
C3_-3_ 1: 3	12.6 ± 1 B	2.39 ± 0.58 B	37 ± 3 A	89.9 ± 22.6 C
**C4 (Coconut coir powder: sand)**	**11 ± 0.7 d**	**1.85 ± 0.02 f**	**21 ± 1 f**	**38.2 ± 1.7 f**
C4_-1_ 3: 1	10.2 ± 1 B	1.55 ± 0.16 B	14 ± 2 B	21.1 ± 3.9 C
C4_-2_ 2: 2	11.6 ± 0.8 A	2.49 ± 0.08 A	24 ± 2 A	58.7 ± 5.5 A
C4_-3_ 1: 3	11.2 ± 0.9 A	1.50 ± 0.09 B	25 ± 1 A	37.5 ± 3.5 B
**C5 (Coconut coir powder: deciduous leaf powder: red soil = 1: 1: 1)**	**11.8 ± 1 c**	**1.97 ± 0.1 ef**	**25 ± 2 e**	**48.6 ± 3.7 e**
**C6 (Coconut coir powder: red soil: sand = 1: 1: 1)**	**14.2 ± 1 a**	**3.56 ± 0.28 a**	**30 ± 5 cd**	**105.9 ± 15.4 ab**
**C7 Single coconut coir powder**	**10.2 ± 0.6 d**	**2.22 ± 0.23 de**	**19 ± 3 f**	**41.9 ± 6 ef**
**C8 Single red soil**	**13.1 ± 0.6 b**	**3.39 ± 0.28 ab**	**31 ± 3 bc**	**104.9 ± 9.1 b**
**C9 Single deciduous leaf powder**	**8 ± 1.1 e**	**0.79 ± 0.11 g**	**14 ± 3 g**	**11.6 ± 3.8 g**
**C10 Single sand**	**13.5 ± 0.9 ab**	**2.34 ± 0.41 d**	**33 ± 2 b**	**76.9 ± 13.3 d**

### Effect of substrate combinations and proportions on the root architecture of *D*. *odorifera*

Total root length, total root surface area, total root volume, and number of root tips and branches showed significant differences (P < 0.05) among groups from different substrate combinations and proportions, but no significant difference (P > 0.05) in the average diameter of roots was observed (Table 2). In general, the mixed substrates may efficiently promote the root growth and development of *D*. *odorifera* than any single substrate. Seedlings from the C6 group had the most excellent performance in total root length, total root surface area, and the total root volume, whereas seedlings from the C3 group had the most number of root tips and branches and the largest total root length compared with those in other groups. Different substrate proportions from different subgroups caused considerable difference in total root length, total root surface area, total root volume, and number of root tips and branches within the same group with same substrate composition. Broadly, the C1_-2_, C2_-2_, and C3_-1_ subgroups had better overall performance in all the above parameters than the other subgroups within the same group and even better than the C6 group. Interestingly, the single substrate of red soil had a balanced and relatively better performance in total root length, total root surface area, and total root volume but poor performance in terms of number of root tips and branches (Fig 2). The single substrate of coconut coir powder, deciduous leaf powder, and sand substantially inhibited the root system and the growth and development of *D*. *odorifera*. Thus, culture substances consisting of sand, coconut coir powder, and red soil could improve the root system and the growth and development of *D*. *odorifera*. Subgroups C1_-2_, C2_-2_, and C3_-2_ could remarkably promote development of root systems.

**Table 2:** Effect of substrate combinations and proportions on the development of *D*. *odorifera* root systems. Different lowercase letters in the same column indicate the significant difference among different substrate combinations (P< 0.05). Different uppercase letters in the same column indicate the significant difference among the same substrate with different proportions (P< 0.05).

Treatment group	Total root length (cm)	Total surface area (cm^2^)	Total root volume (cm^3^)	Average diameter (mm)	Number of root tip	Number of shoot branches
**C1 (Coconut coir powder**: **red soil)**	**104 ± 5.2 b**	**45.77 ± 9.29 ab**	**1.74 ± 0.76 ab**	**1.37 ± 0.25 ab**	**606 ± 115 bc**	**679 ± 113 abc**
C1_-1_ 3: 1	86.5 ± 17.3 B	32.57 ± 12.97 B	1.1 ± 0.82 A	1.21 ± 0.49 A	564 ± 223 A	627 ± 177 AB
C1_-2_ 2: 2	122.5 ± 14.6 A	60.59 ± 24.69 A	2.57 ± 1.95 A	1.54 ± 0.47 A	631 ± 169 A	920 ± 395 A
C1_-3_ 1: 3	103.1 ± 8.7 B	44.16 ± 9.09 AB	1.55 ± 0.57 A	1.36 ± 0.27 A	624 ± 190 A	490 ± 142 B
**C2 (Deciduous leaf powder: red soil)**	**97.9 ± 4.4 bc**	**48.78 ± 6.91 ab**	**2.04 ± 0.65 a**	**1.60 ± 0.25 ab**	**446 ± 63 de**	**613 ± 45 abc**
C2_-1_ 3: 1	66.0 ± 7.9 C	28.87 ± 4.75 B	1.01 ± 0.21 B	1.39 ± 0.07 A	294 ± 77 B	445 ± 149 A
C2_-2_ 2: 2	122.9 ± 13.4 A	65.43 ± 14.91 A	2.95 ± 1.44 A	1.84 ± 0.57 A	492 ± 182 A	734 ± 273 A
C2_-3_ 1: 3	105 ± 9.1 B	52.03 ± 15.32 A	2.17 ± 1.15 AB	1.57 ± 0.42 A	553 ± 146 A	660 ± 166 A
**C3 (Red soil: sand)**	**119.2 ± 11.5 a**	**48.52 ± 9.32 ab**	**1.67 ± 0.62 ab**	**1.34 ± 0.18 ab**	**792 ± 136 a**	**805 ± 125 a**
C3_-1_ 3: 1	118.9 ± 12.8 AB	57.42 ± 16.08 A	2.4 ± 1.53 A	1.56 ± 0.53 A	752 ± 200 AB	905 ± 313 A
C3_-2_ 2: 2	143.5 ± 24 A	52.29 ± 14.38 AB	1.54 ± 0.6 A	1.15 ± 0.17 A	1015 ± 28 A	895 ± 174 A
C3_-3_ 1: 3	95.2 ± 16.2 B	35.85 ± 7.49 B	1.08 ± 0.29 A	1.32 ± 0.11 A	610 ± 155 B	617 ± 252 A
**C4 (Coconut coir powder: sand)**	**71.4 ± 8.6 e**	**29.20 ± 5.34 c**	**1.05 ± 0.44 bc**	**1.29 ± 0.20 ab**	**367 ± 69 e**	**502 ± 105 cd**
C4_-1_ 3: 1	46.3 ± 12.4 C	18.35 ± 5.16 B	0.59 ± 0.2 B	1.26 ± 0.17 A	184 ± 55 B	364 ± 98 A
C4_-2_ 2: 2	73.1 ± 14.4 B	24.57 ± 8.3 B	0.68 ± 0.36 B	1.06 ± 0.2 A	401 ± 87 A	569 ± 121 A
C4_-3_ 1: 3	94.9 ± 11.3 A	44.68 ± 12.37 A	1.88 ± 1.28 A	1.55 ± 0.6 A	515 ± 234 A	572 ± 207 A
**C5 (Coconut coir powder: deciduous leaf powder: red soil = 1: 1: 1)**	**100.3 ± 12.2 b**	**47.39 ± 7.85 ab**	**1.81 ± 0.55 ab**	**1.51 ± 0.21 ab**	**423 ± 108 e**	**582 ± 185 bcd**
**C6 (Coconut coir powder: red soil: sand = 1: 1: 1)**	**130 ± 13.3 a**	**57.72 ± 9.75 a**	**2.05 ± 0.52 a**	**1.19 ± 0.28 b**	**728 ± 108 ab**	**725 ± 219 ab**
**C7 Single coconut coir powder**	**74.1 ± 9.7 de**	**39.77 ± 13.41 bc**	**1.77 ± 1 ab**	**1.68 ± 0.37 a**	**339 ± 48 e**	**548 ± 184 bcd**
**C8 Single red soil**	**101.1 ± 9.3 b**	**45.34 ± 11.16 b**	**1.66 ± 0.62 ab**	**1.41 ± 0.27 ab**	**630 ± 189 bc**	**545 ± 186 bcd**
**C9 Single deciduous leaf powder**	**32.5 ± 9.4 f**	**13.36 ± 3.13 d**	**0.49 ± 0.26 c**	**1.4 ± 0.49 ab**	**127 ± 53 f**	**232 ± 102 e**
**C10 Single sand**	**86.0 ± 9.4 cd**	**33.46 ± 2.82 c**	**1.05 ± 0.17 bc**	**1.44 ± 0.17 ab**	**571 ± 87 cd**	**386 ± 98 de**

### Dry matter accumulation and allocation in *D*. *odorifera* affected by various composite substrates

Different substrate combinations and proportions significantly (P < 0.05) affected the root dry weight (RDW), leaf dry weight (LDW), shoot dry weight (SDW), total dry weight (TDW), SLA, and dry matter allocation (DMA) (Table 3). Among the 10 groups filled with different substrate combinations, the C6 group had the highest RDW and SDW, the C3 group had the best LDW and TDW, and no statistically significant effects (P > 0.05) on RDW, SDW, LDW, and TDW were found among the C1, C2, C3 and C6 groups. The C9 group accumulated the least biomass. Different substrate proportions from different subgroups caused substantial effects on RDW, SDW, LDW, and TDW within the same group with the same substrate composition. For example, the C1_-2_, C2_-2_, and C3_-2_ subgroups had a substantially higher biomass in the above parameters than the other subgroups within the same group, and even better than the C6 group. Interestingly, the single substrate of red soil had a balanced and relatively better performance in biomass accumulation. In addition, the C5 group achieved the maximum values in SLA and DMA, whereas the C9 group obtained the minimum values. The groups with good performance in dry matter accumulation, such as the C1, C2, C3 and C6 groups, had intermediate levels of SLA and DMA, but the effects were not significant. Thus, culture substances consisting of sand, coconut coir powder, and red soil could improve the dry matter accumulation of *D*. *odorifera* seedlings, and subgroups of C1_-2_ and C3_-2_ had the best effects on biomass accumulation.

**Table 3:** Effect of substrate combinations and proportions on the biomass accumulation and allocation of *D*. *odorifera* seedlings. Different lowercase letters in the same column indicate the significant difference among different substrate combinations (P < 0.05). Different uppercase letters in the same column indicate the significant difference among the same substrate with different proportions (P < 0.05).

Treatment group	Root dry weight (mg)	Leaf dry weight (mg)	Shoot dry weight (mg)	Total dry weight (mg)	SLA (cm2/ g)	Dry matter allocation
**C1 (Coconut coir powder: red soil)**	**247.6 ± 23.3 abc**	**860.1 ± 113 abc**	**327.2 ± 36.5 abc**	**1435 ± 144.2 ab**	**103 ± 17 bc**	**0.21 ± 0.01 cd**
C1_-1_ 3: 1	159.2 ± 68.9 B	630.2 ± 154.6 A	278.6 ± 52.7 B	1068 ± 100.2 A	124 ± 33 A	0.18 ± 0.08 A
C1_-2_ 2: 2	384.7 ± 126.4 A	1280.2 ± 641.9 A	477.6 ± 105.3 A	2142.6 ± 152.8 A	135 ± 31 A	0.23 ± 0.03 A
C1_-3_ 1: 3	199 ± 39.8 B	670 ± 130.6 A	225.5 ± 50.1 B	1094.5 ± 320.3 A	102 ± 45 A	0.23 ± 0.02 A
**C2 (Deciduous leaf powder: red soil)**	**245.4 ± 60.8 abc**	**884.4 ± 155.6 abc**	**329.5 ± 44.2 abc**	**1459.3 ± 247.5 ab**	**104 ± 15 bc**	**0.2 ± 0.03 cd**
C2_-1_ 3: 1	152.6 ± 80.4 B	537.3 ± 86.7 B	218.9 ± 39.8 B	908.8 ± 199.3 B	81 ± 16 B	0.2 ± 0.07 A
C2_-2_ 2: 2	338.3 ± 52.7 A	1134.3 ± 139.3 A	424.5 ± 70.2 A	1897.1 ± 191.2 A	101 ± 15 B	0.22 ± 0.02 A
C2_-3_ 1: 3	245.4 ± 57.4 AB	981.7 ± 150.6 AB	344.9 ± 75.3 AB	1572.1 ± 168 A	133 ± 29 A	0.19 ± 0.02 A
**C3 (Red soil: sand)**	**349.8 ± 44.4 a**	**1083.4 ± 136.3 a**	**364.8 ± 69.2 ab**	**1798.1 ± 201.4 a**	**106 ± 11 bc**	**0.24 ± 0.02 bcd**
C3_-1_ 3: 1	391.4 ± 69.9 A	1147.6 ± 291.3 A	364.8 ± 150.7 A	1903.8 ± 179.8 A	131 ± 30 A	0.26 ± 0.02 B
C3_-2_ 2: 2	477.6 ± 86.7 A	1167.5 ± 141.2 A	424.5 ± 126.4 A	2069.6 ± 132.4 A	96 ± 9 B	0.3 ± 0.03 A
C3_-3_ 1: 3	180.4 ± 28.0 B	935.3 ± 340.1 A	305.1 ± 69.9 A	1420.9 ± 150.9 A	97 ± 33 B	0.15 ± 0.02 C
**C4 (Coconut coir powder: sand)**	**269.8 ± 61.6 ab**	**572.7 ± 43.1 bcd**	**276.4 ± 56.4 bc**	**1118.8 ± 129.7 abc**	**68 ± 7 c**	**0.32 ± 0.06 ab**
C4_-1_ 3: 1	139.3 ± 52.7 A	471 ± 141.2 B	265.3 ± 60.8 AB	875.6 ± 121.6 B	47 ± 12 B	0.19 ± 0.06 B
C4_-2_ 2: 2	351.6 ± 96.3 A	378.1 ± 162.9 B	199 ± 91.2 B	928.7 ± 191.1 B	185 ± 87 A	0.58 ± 0.11 A
C4_-3_ 1: 3	318.4 ± 71.8 A	869 ± 41.4 A	364.8 ± 82.9 A	1552.2 ± 71.8 A	44 ± 5 B	0.26 ± 0.07 B
**C5 (Coconut coir powder: deciduous leaf powder: red soil = 1: 1: 1)**	**199 ± 105.3 bcd**	**331.7 ± 268.7 d**	**305.1 ± 69.9 bc**	**835.8 ± 123.5 bc**	**336 ± 34 a**	**0.35 ± 0.15 a**
**C6 (Coconut coir powder: red soil: sand = 1: 1: 1)**	**382.1 ± 143.3 a**	**907.4 ± 487.6 ab**	**461.7 ± 162.6 a**	**1751.2 ± 161.7 a**	**139 ± 73 bc**	**0.3 ± 0.11 abc**
**C7 Single coconut coir powder**	**104.8 ± 21.9 cd**	**437.8 ± 121 cd**	**252.1 ± 50.1 bc**	**794.7 ± 157.7 bc**	**103 ± 33 bc**	**0.16 ± 0.06 d**
**C8 Single red soil**	**238.8 ± 121 abc**	**961.8 ± 174.8 ab**	**351.6 ± 75.3 abc**	**1552.2 ± 165.7 a**	**128 ± 52 bc**	**0.18 ± 0.02 d**
**C9 Single deciduous leaf powder**	**79.6 ± 19.9 d**	**252.1 ± 30.4 d**	**218.9 ± 39.8 c**	**550.6 ± 64 cd**	**49 ± 15 c**	**0.17 ± 0.04 d**
**C10 Single sand**	**132.7 ± 64.0 abcd**	**305.1 ± 116.6 d**	**212.3 ± 23 cd**	**650.1 ± 127.9 c**	**253 ± 69 ab**	**0.27 ± 0.14 abcd**

### Effects of substrate combinations and proportions on the leaf REL and pigment content

Various substrate combinations and proportions significantly (P < 0.05) changed the leaf REL and the contents of chlorophyll a, chlorophyll b, carotenoids, and total chlorophyll in *D*. *odorifera* (Table 4). Among the 10 groups filled with different substrate combinations, the C1, C2, C3, and C6 groups had relatively lower REL and higher levels of chlorophyll a, chlorophyll b, carotenoids, and total chlorophyll. The C1_-2_, C2_-2_, and C3_-2_ subgroups had a substantially lower level of leaf REL but had higher levels of chlorophyll contents than the other subgroups within the same groups and even better than the C6 group. The C2_-2_ subgroup and the C7 group had the lowest and highest levels of leaf REL, respectively. The C2_-2_ and C3_-1_ subgroups had the highest levels, whereas the C9 group had the lowest levels of chlorophyll contents. In addition, the single red soil substrate had balanced and intermediate performance in leaf REL and photosynthetic pigment contents. Thus, culture substances consisting of sand, coconut coir powder, and red soil could increase the pigment content of *D*. *odorifera* leaves.

**Table 4:** Effect of substrate combinations and proportions on the leaf relative electrolyte leakage and pigment contents of *D*. *odorifera*. Different lowercase letters in the same column indicate the significant difference among different substrate combinations (P < 0.05). Different uppercase letters in the same column indicate the significant difference among the same substrate with different proportions (P < 0.05).

Treatment group	REL (%)	Chlorophyll a (μg / g· FW)	Chlorophyll b (μg / g· FW)	Carotenoids (μg / g· FW)	Chlorophyll (μg / g· FW)
**C1 (Coconut coir powder**: **red soil)**	**41.79 ± 1.77 bcd**	**152 ± 10 b**	**52 ± 0 bc**	**42 ± 10 c**	**204 ± 20 b**
C1_-1_ 3: 1	43.12 ± 0.04 A	103 ± 30 B	38 ± 0 B	27 ± 10 B	141 ± 30 B
C1_-2_ 2: 2	39.36 ± 0.04 A	221 ± 30 A	79 ± 10 A	64 ± 20 A	300 ± 40 B
C1_-3_ 1: 3	42.88 ± 0.05 A	132 ± 20 B	38 ± 10 B	36 ± 0 B	170 ± 30 A
**C2 (Deciduous leaf powder: red soil)**	**38.01 ± 3.27 a**	**206 ± 30 a**	**65 ± 10 ab**	**56 ± 10 b**	**271 ± 30 a**
C2_-1_ 3: 1	43.88 ± 0.05 A	178 ± 50 B	56 ± 10 B	56 ± 10 A	235 ± 60 B
C2_-2_ 2: 2	33.69 ± 0.06 B	236 ± 40 A	76 ± 10 A	62 ± 10 A	312 ± 50 A
C2_-3_ 1: 3	36.46 ± 0.01 B	205 ± 20 AB	61 ± 10 AB	50 ± 10 A	266 ± 30 AB
**C3 (Red soil: sand)**	**41.66 ± 1.48 bcd**	**202 ± 20 a**	**65 ± 0 ab**	**44 ± 10 c**	**267 ± 30 a**
C3_-1_ 3: 1	40.29 ± 0.01 B	244 ± 40 A	79 ± 20 A	52 ± 10 A	323 ± 60 A
C3_-2_ 2: 2	38.5 ± 0.05 B	215 ± 40 A	64 ± 20 AB	47 ± 10 AB	279 ± 60 A
C3_-3_ 1: 3	46.2 ± 0.01 A	148 ± 30 B	50 ± 20 B	34 ± 10 B	199 ± 40 B
**C4 (Coconut coir powder: sand)**	**44.05 ± 0.99 abc**	**113 ± 10 c**	**37 ± 10 cd**	**31 ± 0 d**	**150 ± 10 c**
C4_-1_ 3: 1	43.25 ± 0 A	105 ± 20 B	35 ± 10 AB	30 ± 10 A	140 ± 20 B
C4_-2_ 2: 2	44.23 ± 0.01 A	154 ± 20 A	45 ± 0 A	44 ± 10 A	198 ± 30 A
C4_-3_ 1: 3	44.67 ± 0.03 A	82 ± 10 B	31 ± 0 B	19 ± 0 C	113 ± 10 B
**C5 (Coconut coir powder: deciduous leaf powder: red soil) = 1: 1: 1**	**45.03 ± 0.02 abc**	**155 ± 30 b**	**49 ± 10 cd**	**47 ± 10 bc**	**204 ± 30 b**
**C6 (Coconut coir powder: red soil: sand = 1: 1: 1)**	**40.36 ± 0.01 cd**	**218 ± 20 a**	**62 ± 10 ab**	**66 ± 10 a**	**280 ± 30 a**
**C7 Single coconut coir powder**	**47.63 ± 0.02 a**	**127 ± 20 bc**	**61 ± 10 ab**	**21 ± 10 e**	**188 ± 30 bc**
**C8 Single red soil**	**42.73 ± 0.04 abcd**	**208 ± 30 a**	**68 ± 10 ab**	**52 ± 10 bc**	**276 ± 50 a**
**C9 Single deciduous leaf powder**	**46.01 ± 0.09 ab**	**68 ± 10 d**	**30 ± 0 d**	**17 ± 0 e**	**97 ± 10 d**
**C10 Single sand**	**41.79 ± 0.01 bcd**	**200 ± 50 a**	**81 ± 40 a**	**46 ± 10 c**	**281 ± 80 a**

### Effects of substrate combinations and proportions on the MDA content, SOD activity, and soluble sugar contents of *D*. *odorifera* root systems

Various substrate combinations and proportions significantly (P < 0.05) changed the MDA, SOD, and sugar contents of *D*. *odorifera* root systems (Table 5). Among the 10 groups filled with different substrate combinations, the C9 group had the highest levels of MDA content and SOD activity. The C1, C2, C3, and C6 groups had relatively lower levels of MDA content and SOD activity. The C6 group had the lowest levels in these parameters. The C1_-2_, C2_-2_, and C3_-2_ subgroups had a remarkable lower levels of MDA content and SOD activity than the other subgroups within the same groups but almost the as those of the C6 group. In general, the C3_-1_ subgroup had the highest level and the C5 group had the lowest level of sugar contents. The C1_-2_ and C2_-2_ and the C6 groups had remarkably higher sugar contents than the other groups, whereas the C5, C7, and C9 groups had substantially lower sugar contents. In addition, the single red soil substrate had balanced and intermediate effects on the MDA content, SOD activity, and sugar content of *D*. *odorifera* root systems. Thus, culture substances consisting of sand, coconut coir powder, and red soil, particularly C3_-1_ subgroup, could improve the physiological traits of *D*. *odorifera* roots.

**Table 5:** Effect of substrate combinations and proportions on malondialdehyde content, superoxide dismutase activity, and sugar content of *D*. *odorifera* root systems. Different lowercase letters in the same column indicate the significant difference among different substrate combinations (P < 0.05). Different uppercase letters in the same column indicate the significant difference among the same substrate with different proportions (P < 0.05).

Treatment group	MDA contents (nmol / g · FW)	SOD activities (U / g · FW)	Sugar contents (μg / g · FW)
**C1 (Coconut coir powder**: **red soil)**	**398.5 ± 8.0 e**	**194.2 ± 3.0e**	**3.77 ± 0.08 d**
C1_-1_ 3: 1	430.4 ± 11.9 A	228.5 ± 0.7A	3.86 ± 0.05 B
C1_-2_ 2: 2	309.3 ± 0 B	150.7 ± 5.7C	4.35 ± 0.27 A
C1_-3_ 1: 3	455.8 ± 35.7 A	203.4 ± 2.7B	3.1 ± 0.09 C
**C2 (Deciduous leaf powder: red soil)**	**392.7 ± 8.3 e**	**194.4 ± 0.8e**	**4.07 ± 0.06 bc**
C2_-1_ 3: 1	437.1 ± 23.8 A	233.9 ± 0.9 A	3.82 ± 0.06 B
C2_-2_ 2: 2	313.2 ± 32.4 B	164 ± 0.7 C	4.5 ± 0.14 A
C2_-3_ 1: 3	427.9 ± 33.4 A	185.5 ± 0.8 B	3.88 ± 0.08 B
**C3 (Red soil: sand)**	**394.2 ± 24.1 e**	**169.2 ± 1.4 g**	**4.13 ± 0.08 b**
C3_-1_ 3: 1	404 ± 9.1 A	157.3 ± 0.6 B	5.36 ± 0.09 A
C3_-2_ 2: 2	315.3 ± 5.5 B	140.2 ± 2 C	3.49 ± 0.27 B
C3_-3_ 1: 3	463.2 ± 68.6 A	210 ± 1.4 A	3.54 ± 0.26 B
**C4 (Coconut coir powder: sand)**	**725.4 ± 40.0 b**	**181.5 ± 2.3 f**	**3.65 ± 0.07 de**
C4_-1_ 3: 1	616.3 ± 19.3 B	234.8 ± 2.7 A	4.04 ± 0.24 A
C4_-2_ 2: 2	649.7 ± 4.7 B	153 ± 0.8 B	3.74 ± 0.06 B
C4_-3_ 1: 3	910.1 ± 98.6 A	156.7 ± 4.9 B	3.18 ± 0.02 C
**C5 (Coconut coir powder: deciduous leaf powder: red soil) = 1: 1: 1**	**500.1 ± 35.5 c**	**219.9 ± 2.8 d**	**3.09 ± 0.14 f**
**C6 (Coconut coir powder: red soil: sand = 1: 1: 1)**	**309 ± 13.2 f**	**124.7 ± 0.6 h**	**4.51 ± 0.02 a**
**C7 Single coconut coir powder**	**807.7 ± 0 a**	**356.4 ± 7.8 b**	**3.48 ± 0.07 ef**
**C8 Single red soil**	**441.4 ± 0 de**	**226.8 ± 2 c**	**3.95 ± 0.05 c**
**C9 Single deciduous leaf powder**	**837.2 ± 73.5 a**	**413.8 ± 0 a**	**3.62 ± 0.06 de**
**C10 Single sand**	**475.7 ± 39.2 cd**	**197.8 ± 0.7 e**	**3.97 ± 0.15 c**

### Comprehensive evaluation of *D*. *odorifera* seedlings affected by various substrate combinations and proportions

A smaller value of ∑P_*i*_^2^ indicates a better effect in Euclid's model. In general, the multidimensional space analysis showed that the groups containing red soil had better performance than the groups without red soil, and appropriate proportions of coconut coir powder, deciduous leaf powder, and sand could improve the performance of *D*. *odorifera* in red soil (Table 6). The comprehensive analysis showed that the C1_-2_, C2_-2_, C3_-1_, and C3_-2_ subgroups and the C6 group are the top five groups in terms of performance in most of the given parameters. The C1_-2_ subgroup filled with equal volumes of coconut coir and red soil had the best overall performance in the comprehensive evaluation, but the gaps were small among the top five groups. The C8 group filled with red soil only ranked seventh in the comprehensive evaluation because of its balanced performance in most parameters but was inferior to the top five groups in terms of overall performence. The C4 group, including the three subgroups filled with coconut coir and sand had a worse performance in the comprehensive evaluation compared with the other groups containing the red soil component. The C7 and C9 groups filled solely with coconut coir powder and deciduous leaf powder, respectively, had the worst performance in the comprehensive evaluation (especially the single deciduous leaf powder substrate).

**Table 6:** Comprehensive evaluation of different substrate combinations and proportions via multi-vector coordinates.

Treatment group	∑P_*i*_^2^	Rank
C1_-1_ Coconut coir powder: red soil = 3: 1	5.112	12
C1_-2_ Coconut coir powder: red soil = 2: 2	0.418	1
C1_-3_ Coconut coir powder: red soil = 1: 3	4.332	9
C2_-1_ Deciduous leaf powder: red soil = 3: 1	5.656	13
C2_-2_ Deciduous leaf powder: red soil = 2: 2	0.700	3
C2_-3_ Deciduous leaf powder: red soil = 1: 3	1.646	6
C3_-1_ Red soil: sand = 3: 1	0.542	2
C3_-2_ Red soil: sand = 2: 2	0.895	5
C3_-3_ Red soil: sand = 1: 3	3.723	8
C4_-1_ Coconut coir powder: sand = 3: 1	8.995	16
C4_-2_ Coconut coir powder: sand = 2: 2	5.896	14
C4_-3_ Coconut coir powder: sand = 1: 3	8.084	15
C5 Coconut coir powder: deciduous leaf powder: Red soil = 1: 1: 1	4.844	11
C6 Coconut coir powder: red soil: sand = 1: 1: 1	0.815	4
C7 Single coconut coir powder	11.150	17
C8 Single red soil	2.451	7
C9 Single deciduous leaf powder	18.085	18
C10 Single sand	4.598	10

## Discussion

Some solid wastes, such as rice coir biochar, almond shells, pineal compost, waste wood, and pine bark, can be used as culture materials to improve germination rate, seedling growth and root development [13, 14, 28] by improve the soil pH and aeration, increasing cation exchange capacity and water-retention capacity, and even providing nutrients [13, 28, 29]. In the present study, the basic physical and chemical properties of culture substances evidently varied when single red soil was mixed with coconut coir powder and sand. For example, the single red soil used in this study had pH 4.69, 0.61 g/kg total nitrogen, 0.18 g/kg total phosphorus, 7.11 g/kg organic matter, and 1.65 g/kg organic carbon. However, the culture substance consisting of equal volumes of coconut coir powder, red soil, and sand had pH 6.23, 1.77 g/kg total nitrogen, 0.64 g/kg total phosphorus, 58.01 g/kg organic matter, 33.65 g/kg organic carbon. Coconut coir and the fallen leaves of *F*. *elastica* possess a large amount of fibers, abundant nutrients, and intense physical elasticity, which can be used to improve the structure, porosity, organic matter content, and fertility of soil and thus can improve the environment for plant growth and development [9, 10, 30, 31]. Therefore, the results of the present study provide the answer to the first research question that coconut coir and the fallen leaves of *F*. *elastica* (typical solid wastes in Hainan Province) could be used as culture substrates to improve the growth and development of *D*. *odorifera* in red soil. The rational utilization of coconut coir and fallen leaves is also favorable for environmental protection.

Several coordinated morphological, physiological, and biochemical responses occur when a plant is exposed to stress [19, 20, 24]. In this study, nutrition deficiency and poor ventilation status were the two main abiotic stresses caused by the different combinations and proportions of culture substrates for *D*. *odorifera* seedlings. Poor performances under stressful environments were evaluated on the basis of the appearance of yellow leaves, observation of poor seedling growth and development, and root architecture, and attainment of low levels of chlorophyll and sugar contents, low ratios of dry matter accumulation and dry matter allocation, and high levels of leaf REL and root MDA [32–34]. Conversely, the excellent performances under desirable growth environments were evaluated on the basis of opposite trends in these parameters.

The above- and below-ground growth and development of seedlings depend not only on the physical properties of culture matrixes such as pH values and porosity, but also on soil fertility. The substrate combinations caused remarkable differences in all the parameters analyzed. The poor growth and development of *D*. *odorifera* in the C5, C7, and C9 groups might have resulted from nutrients deficiency due to the absence of red soil. According to Liebig’s law of the minimum, the factor with the minimum amount is the key limiting factor. In this case, nutrients were the key factors limiting the growth and development of *D*. *odorifera* in the absence of red soil. Membrane permeability (REL), membrane lipid peroxidation (MDA), and antioxidant enzymatic systems would positively respond when plants are exposed to biotic or abiotic stresses [20, 22, 35]. The higher levels of leaf REL, root MDA contents, and root SOD activities in *D*. *odorifera* from the groups without red soil suggested that the seedlings experienced nutrient deficiency, which resulted in poor seedling growth and root architecture and low dry matter accumulation and pigment content. Seedlings in the group C8 cultured solely with red soil had balanced performance in most of the traits analyzed but were inferior to seedlings in groups cultured with mixtures containing red soil. This phenomenon suggested that the physical properties of red soil should be improved for the establishment of *D*. *odorifera* seedlings in spite of its abundant nutrients. Thus, the answer to the second research question was that the seedlings growth and root systems of *D*. *odorifera* under single substrates were inferior to those under mixed substrates.

Some exogenous solid wastes can improve soil environment; However, the proper combinations and proportions of cultures are the key factors to promote plant growth and development. For example, the mixture of perlite and rice coir biochar is superior to single cultivation substrate in increasing the development and yield of cabbage, dill, and red lettuce [9, 13, 14]. Coconut coir powder, deciduous leaf powder, and sand may improve the red soil’s pH and porosity, which in turn enhance the soil’s breathing ability. However, appropriate combinations and proportions among them are essential to improve the growth and development of *D*. *odorifera* seedlings [10, 30, 31]. Among the 10 groups filled with different substrates, seedlings from the C6 group cultured by equal volumes of coconut coir powder, red soil, and sand had the best performance in the above- and below-ground morphological growth and development attributes. Moreover the dry matter accumulation and allocation, pigment content, and root physiological traits, especially development of root nodules, were also enhanced. The substrate proportions from different subgroups within the C1, C2, and C3 groups remarkably improved the overall performance of *D*. *odorifera* seedlings. The performances of seedlings from the C1, C2, and C3 groups were inferior to those of the C6 group because the lower performances of some subgroups within the same group affected their total mean values. In fact, the comprehensive performance and evaluation of seedlings from the C1_-2_ (cultured by equal volumes of coconut coir and red soil), C3_-1_ (cultured by threefold volumes red soil and sand), and C2_-2_ (cultured by equal volumes of deciduous leaf powder and red soil) subgroups were remarkably superior to that of the other subgroups cultured by various proportions of the same substrates within the same group and were even superior to the performance of seedlings from the C6 group. The excellent performance of these seedlings mainly resulted from the appropriate composition of different substrates. Therefore, the answer to the third research question was that the ideal combinations and proportions of culture substrates came from the C1-_2_, C3-_1_, C2-_2_, and C3-_2_ subgroups and C6 group, which are suitable for *D*. *odorifera* seedlings in terms of plant growth, root system and root nodule development, leaf pigment synthesis, and root physiobiochemical characteristics.

## Conclusion

In general, an appropriate proportional amount of coconut coir, deciduous leaf powder, and sand may improve the overall performance of *D*. *odorifera* seedlings in red soil. The ideal substrate composite for *D*. *odorifera* seedlings came from the C1_-2_, C3_-1_, C2_-2_, and C3-_2_ subgroups and C6 group. The results confirmed our hypothesis that the growth, root system development, and physiological characteristics of *D*. *odorifera* differentially respond to the same optimal culture substances. Subgroups C1_-2_ (coconut coir/red soil = 2/2, v/v) and C3_-2_ (red soil/sand = 2/2) exhibited the best effects on plant growth and biomass accumulation. Subgroups C1_-2_, C2_-2_ (deciduous leaf powder/red soil = 2/2), and C3_-2_ could substantially improve root system development. The C6 group (coconut coir/red soil/sand = 1/1/1) remarkably promoted root nodule development. The C3_-1_ subgroup (red soil/sand = 3/1) showed the best effects on physiological characteristics. On the basis of the comprehensive evaluation of Euclid’s multidimensional space mathematical model, the suitable substrate combinations were C1_-2_, followed by C3_-1_, and then C2_-2_. This study provides a scientific basis for the production of healthy seedling cultures of *D*. *odorifera* and a rational utilization of solid wastes such as coconut coir and the deciduous leaves of *F*. *elastica*.
